# Rationale and Design of Assessing the Effectiveness of Short-Term Low-Dose Lithium Therapy in Averting Cardiac Surgery-Associated Acute Kidney Injury: A Randomized, Double Blinded, Placebo Controlled Pilot Trial

**DOI:** 10.3389/fmed.2021.639402

**Published:** 2021-06-14

**Authors:** Sairah Sharif, Bohan Chen, Pamela Brewster, Tian Chen, Lance Dworkin, Rujun Gong

**Affiliations:** ^1^Division of Critical Care Medicine, St Francis Hospital, New York, NY, United States; ^2^Division of Kidney Disease and Hypertension, Department of Medicine, Rhode Island Hospital, Brown University School of Medicine, Providence, RI, United States; ^3^Division of Nephrology, Department of Medicine, University of Toledo Medical Center, Toledo, OH, United States; ^4^Department of Mathematics and Statistics, The University of Toledo, Toledo, OH, United States

**Keywords:** cardiac surgery associated acute kidney injury, lithium, glycogen synthase kinase 3β, acute kidney injury, cardiopulmonary bypass surgery

## Abstract

**Background:** Burgeoning pre-clinical evidence suggests that therapeutic targeting of glycogen synthase kinase 3β (GSK3β), a convergence point of multiple cellular protective signaling pathways, confers a beneficial effect on acute kidney injury (AKI) in experimental models. However, it remains unknown if GSK3β inhibition likewise mitigates AKI in humans. Cardiac surgery associated acute kidney injury (CSA-AKI) poses a significant challenge for clinicians and currently the only treatment available is general supportive measures. Lithium, an FDA approved mood stabilizer, is the best-known GSK3β inhibitor and has been safely used for over half a century as the first line regimen to treat bipolar affective disorders. This study attempts to examine the effectiveness of short term low dose lithium on CSA-AKI in human patients.

**Methods/Design:** This is a single center, prospective, randomized, double blinded, placebo controlled pilot study on patients undergoing cardiac surgery with cardiopulmonary bypass. Patients will be randomized to receive a small dose of lithium or placebo treatment for three consecutive days. Renal function will be measured via creatinine as well as novel AKI biomarkers. The primary outcome is incidence of AKI according to Acute Kidney Injury Network (AKIN) criteria, and secondary outcomes include receipt of new dialysis, days on dialysis, days on mechanical ventilation, infections within 1 month of surgery, and death within 90 days of surgery.

**Discussion:** As a standard selective inhibitor of GSK3β, lithium has been shown to exert a beneficial effect on tissue repair and regeneration upon acute injury in multiple organ systems, including the central nervous system and hematopoietic system. In experimental AKI, lithium at small doses is able to ameliorate AKI and promote kidney repair. Successful completion of this study will help to assess the effectiveness of lithium in CSA-AKI and could potentially pave the way for large-scale randomized trials to thoroughly evaluate the efficacy of this novel regimen for preventing AKI after cardiac surgery.

**Trial Registration:** This study was registered prospectively on the 17th February 2017 at ClinicalTrials.gov (NCT03056248, https://clinicaltrials.gov/ct2/show/NCT03056248?term=NCT03056248&draw=2&rank=1).

## Introduction

Cardiac surgery-associated acute kidney injury (CSA-AKI) is a significant problem. The prevalence varies from 0.3 to 22.9% depending on the definition of acute kidney injury (AKI) (1). In a study of 2,222 patients undergoing elective cardiopulmonary bypass (CPB) surgery with or without valvular surgery, 7.7% had AKI, and 1.4% required dialysis (2). Another prospective study by Ostermann et al. of 2,337 patients undergoing cardiac surgery, 2.7% needed continuous renal replacement therapy (3). The etiopathogenesis of AKI in this population is multifactorial (4). Factors associated with AKI in such patients include patient comorbids, procedure related factors, exogenous nephrotoxic agents, hemodynamic insults and genetic factors (5). Mechanism of AKI includes increased afferent arteriolar vasoconstriction leading to ischemic injury. In addition, CPB induces a proinflammatory state, intravascular hemolysis and hemoglobinuria. Although pulsatile flow overall has improved inflammatory response due to reduced leukocyte adherence, in some studies there is no difference in AKI incidence (6). In the initial course the kidney function may still be intact. However, if there is prolonged ischemic, oxidative, and inflammatory injury, further damage will occur leading to renal tubular cell disruption.

CSA-AKI has significant clinical consequences. It is an independent predictor of mortality, morbidity, increased length of hospitalization and costs (7, 8). In a study of over thirty-one thousand patients it was found that a reduction of glomerular filtration rate beyond 30% was associated with 4 times the mortality risk (9). Another retrospective study by Thakar et al. showed that incidence of infections after cardiac surgery was 1.6% in patients without renal failure, 23.7% in patients with renal failure not requiring dialysis, and 58.5% in patients with renal failure requiring dialysis (10). Moreover, these patients were also at increased risk of end stage renal disease in the future (11–13). In another study where AKI was classified according to RIFLE criteria, it was found that survival post cardiac surgery was associated with varying stages of AKI, with 51% at the risk of AKI, 42% having kidney injury, and 26% having kidney failure (14).

Various pharmacologic approaches have been tried in hope of preventing early CSA-AKI, including diuretics, vasodilators, and anti-inflammatory drugs. For instance, nesiritide was studied in a retrospective cohort study in adult non-transplant cardiac surgery patients. It showed reduction in dialysis and death with no statistical significance (15). In a prospective study fenoldopam and N-acetylcysteine were found to improve creatinine clearance by 6.5 and 7.5 ml, respectively, with no change in length of stay in hospital or intensive care unit (16). However, other studies have shown contrary results (17, 18). Sodium bicarbonate, statins and mannitol have been tried but did not demonstrate consistent and significant effects on improvement of AKI (19, 20). The above mentioned therapeutic preventive strategies lack high quality evidence to support their use and are not standard of care (21). Thus, the current best therapy for CSA-AKI is still limited to optimizing patient hemodynamics, supportive care, and renal replacement therapy when indicated (22, 23).

**Figure 1:**
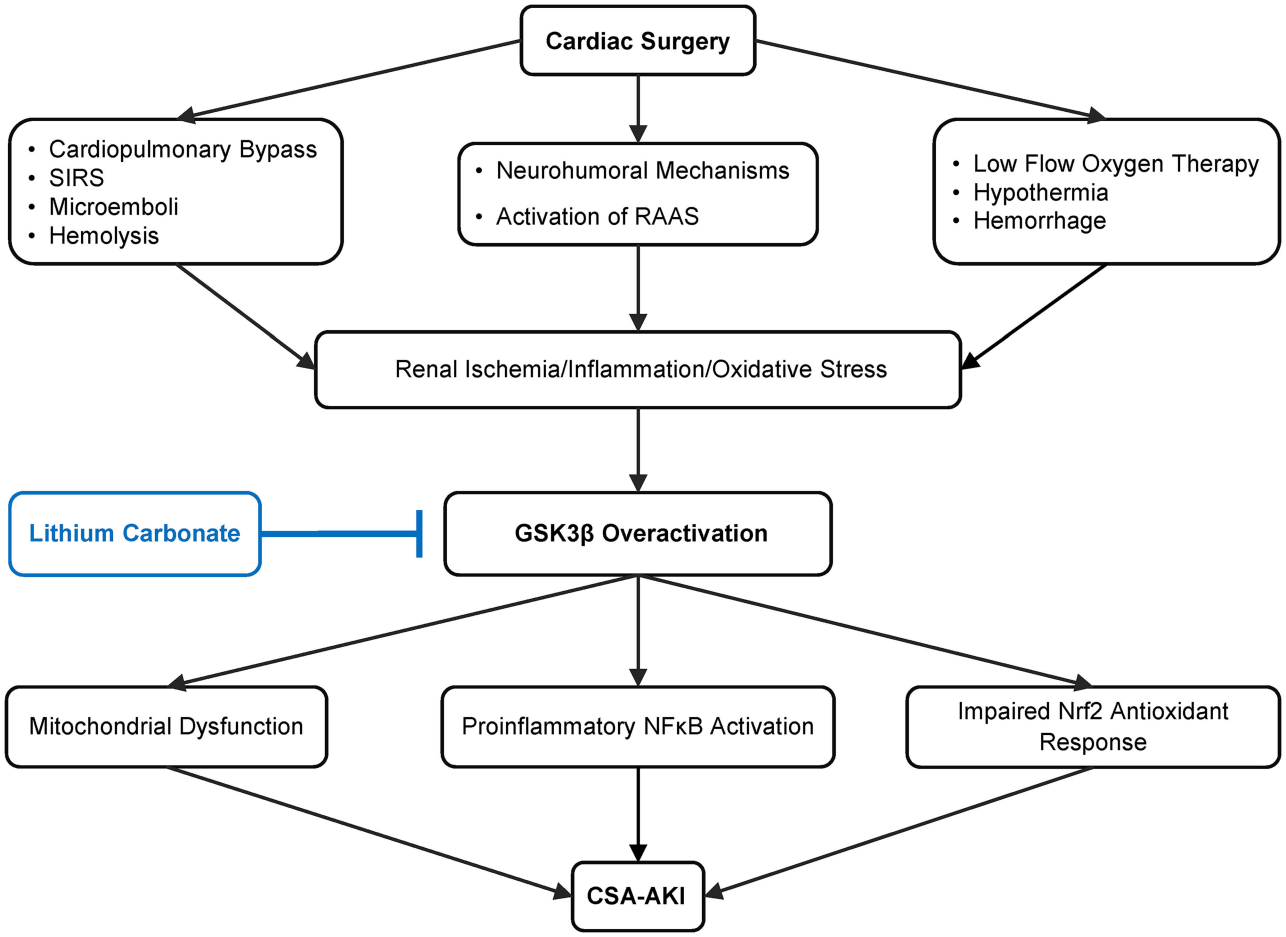
Schematic diagram depicting pathways leading to cardiovascular surgery-associated acute kidney injury (CSA-AKI) and role of GSK3β in the pathogenesis. There are multiple, complicated pathways mediating CSA-AKI. Cardiovascular surgery is a state of low blood flow, there is hypothermia, blood loss, activation of renin angiotensin aldosterone system (RAAS). Additionally due to CPB there is hemolysis, systemic inflammatory response syndrome (SIRS) and microemboli formation. Together these lead to reduced renal perfusion, ischemia, increase in oxidative and inflammatory injury, which in turn overactivate GSK3β. GSK3β increases proinflammatory NFκB activation, potentiates mitochondrial dysfunction as well as impairs Nrf2 antioxidant response, culminating in CSA-AKI. As an FDA-approved mood stabilizer, lithium is a standard and effective inhibitor of GSK3β and is able to ameliorate diverse types of AKI in pre-clinical models. Its efficacy in preventing CSA-AKI will be tested by this prospective, randomized, double blinded, placebo-controlled pilot clinical trial.

AKI in this setting is thus an extremely complex process involving multiple pathophysiologic pathways, among which glycogen synthase kinase 3β (GSK3β) has emerged as a key factor mediating the pathogenesis of AKI. As a highly conserved serine/threonine protein kinase that is ubiquitously expressed, GSK3β was originally found in early 1980s to be a pivotal transducer of the insulin signaling pathway *via* phosphorylating glycogen synthase and thereby regulating glucose metabolism and the biogenesis of glycogen (24, 25). But recently, a growing body of evidence suggests that GSK3β is also centrally involved in a large number of other cellular signaling pathways, including Wnt/β-catenin, NFκB, CREB pathways and cytoskeleton regulation (26, 27). Of the many signaling cascades, GSK3β is situated at the nexus of the reperfusion injury salvage kinase pathway (involving PI3K/Akt signaling) and the survivor activating factor enhancement pathway (involving JAK/STAT3 signaling) and thus become a convergence point of protective signaling (28, 29). In concordance, GSK3β has been implicated in organ injury, repair and regeneration. Therapeutic targeting of GSK3β has demonstrated a potent beneficial action in numerous animal models of acute organ injury, including AKI (Figure 1). While the highly selective small molecule inhibitors of GSK3β have exhibited promising therapeutic effects in pre-clinical studies, they may take years or even decades to go through human trials and be approved for use in patients (30).

Luckily, there are some Food and Drug Administration (FDA)-approved drugs with GSK3β inhibitory activities. Lithium is the best-known inhibitor of GSK3β and has been used as standard selective inhibitor of GSK3β for basic science research for decades. In addition, lithium is a US FDA–approved mood stabilizer, which has been used for nearly seven decades as first line agent to treat bipolar affective disorders in adults and children (31, 32). Indeed, lithium has shown powerful protective effect on a multitude of models of acute organ injury, including AKI, and this beneficial action has been attributable to GSK3β inhibition, which subsequently prevents cellular apoptosis and promotes cellular repair and repopulation (see **Figure 3**). It has been shown to have a positive effect on tissue repair and regeneration in both the central nervous system and hematopoietic system. It causes leukocytosis (33) and increased gray matter in humans (34). In a study by Bao et al. in mouse models of cisplatin as well as ischemia/reperfusion-induced AKI, there was a reduction in serum creatinine (SCr) in lithium treated mice as compared to controls. Lithium was found to promote repair of tubular cells hence improvement of AKI in murine model (35). Moreover, low dose lithium also has a demonstrable anti-proteinuric and renal regenerative effect (36).

In this context, since lithium inhibing GSK3β, has shown to promote renal tubular epithelial survival and recovery of kidney function in pre-clinical studies (35), it is tempting to speculate that therapeutic targeting of GSK3β by short term low dose lithium treatment may protect against AKI in man. This pilot study aims to explore this property of lithium in patients with CSA-AKI. There is a dire need of an efficacious pharmacotherapy in the area of AKI. Successful completion of this study may pave the way to establish a novel therapeutic strategy to reduce the incidence of CSA-AKI.

## Methods

### Study Design

The Lithium in cardiac surgery-associated acute kidney injury (LiCS-AKI) study aims to determine if low dose lithium will impact renal function in patients undergoing elective cardiac surgery requiring CPB. The study is designed as a single center, prospective, randomized, control study where patients undergoing cardiac surgery requiring CPB will be randomized into two groups, namely intervention and placebo groups. The intervention group will receive low-dose oral lithium treatment on three consecutive days while the placebo group will get placebo for the same number of days. As AKI rates are lower in cardiovascular surgery without CPB (37), inclusion of patients without CPB will thus add a confounding variable in the interpretation of data. To overcome this variable, much larger sample sizes of patients would be required. As such, patients undergoing cardiac surgery without CPB will not be included.

### Study Setting

The study will take place in adult inpatient units, including telemetry, regular floor, intensive care units, coronary care units as well as outpatient cardiothoracic clinics. Patients will be identified by chart review and referral from cardiothoracic team. They will be explained about the study and informed consent will be taken. There is no financial compensation for the patients and there will be no advertisement regarding the study.

### Intervention

**Figure 2:**
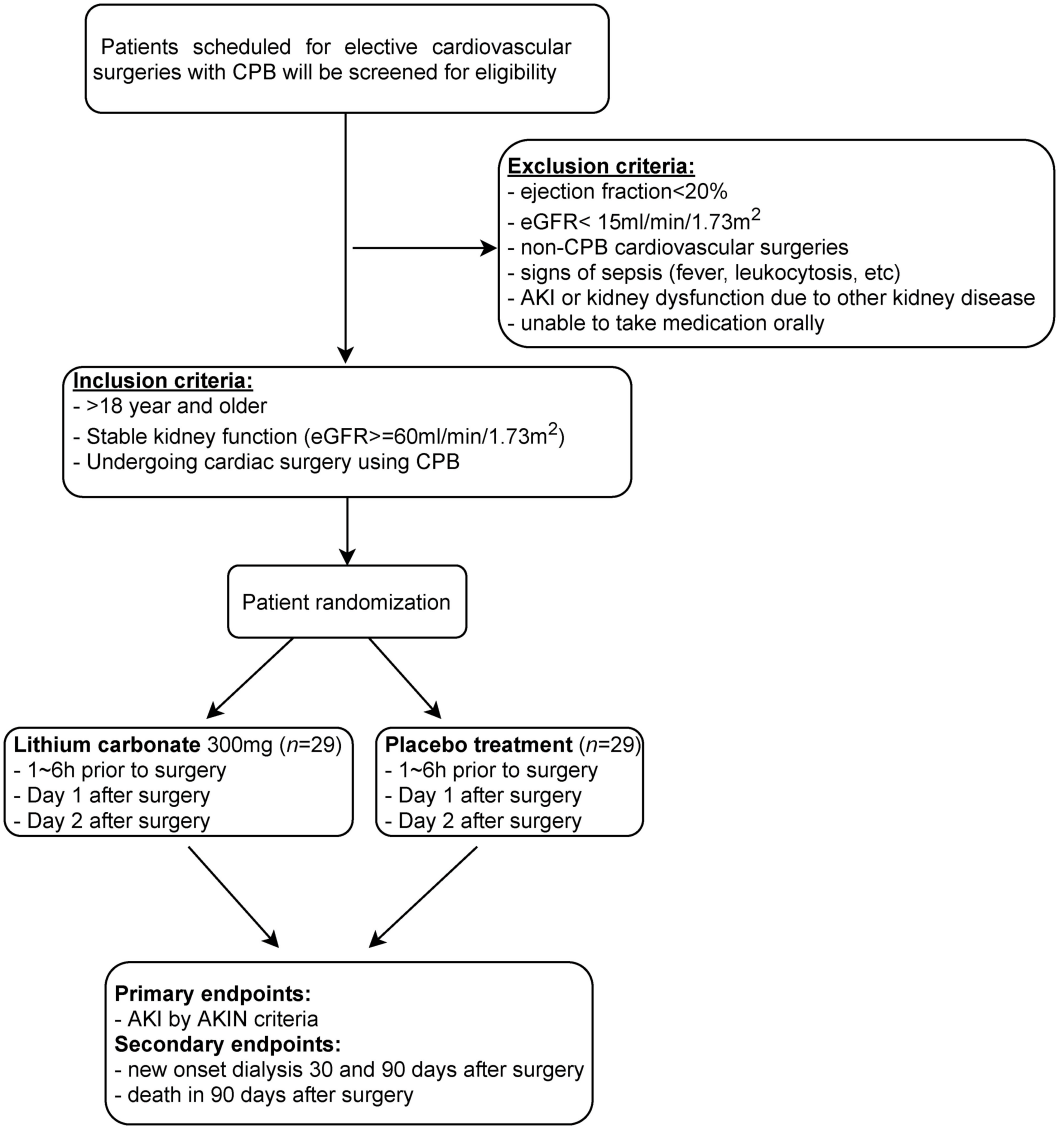
Flow diagram depicting the design of this single center, randomized, double blinded, placebo controlled pilot trial. Patients scheduled to undergo cardiovascular surgeries with cardiopulmonary bypass (CPB) will be subjected to eligibility assessment for inclusion in this trial to test the potential of short-term use of microdose lithium for protecting against CSA-AKI. This will include patients in the emergency department, general floors, critical care units, coronary care unit and step down/ telemetry units.

**Figure 3:**
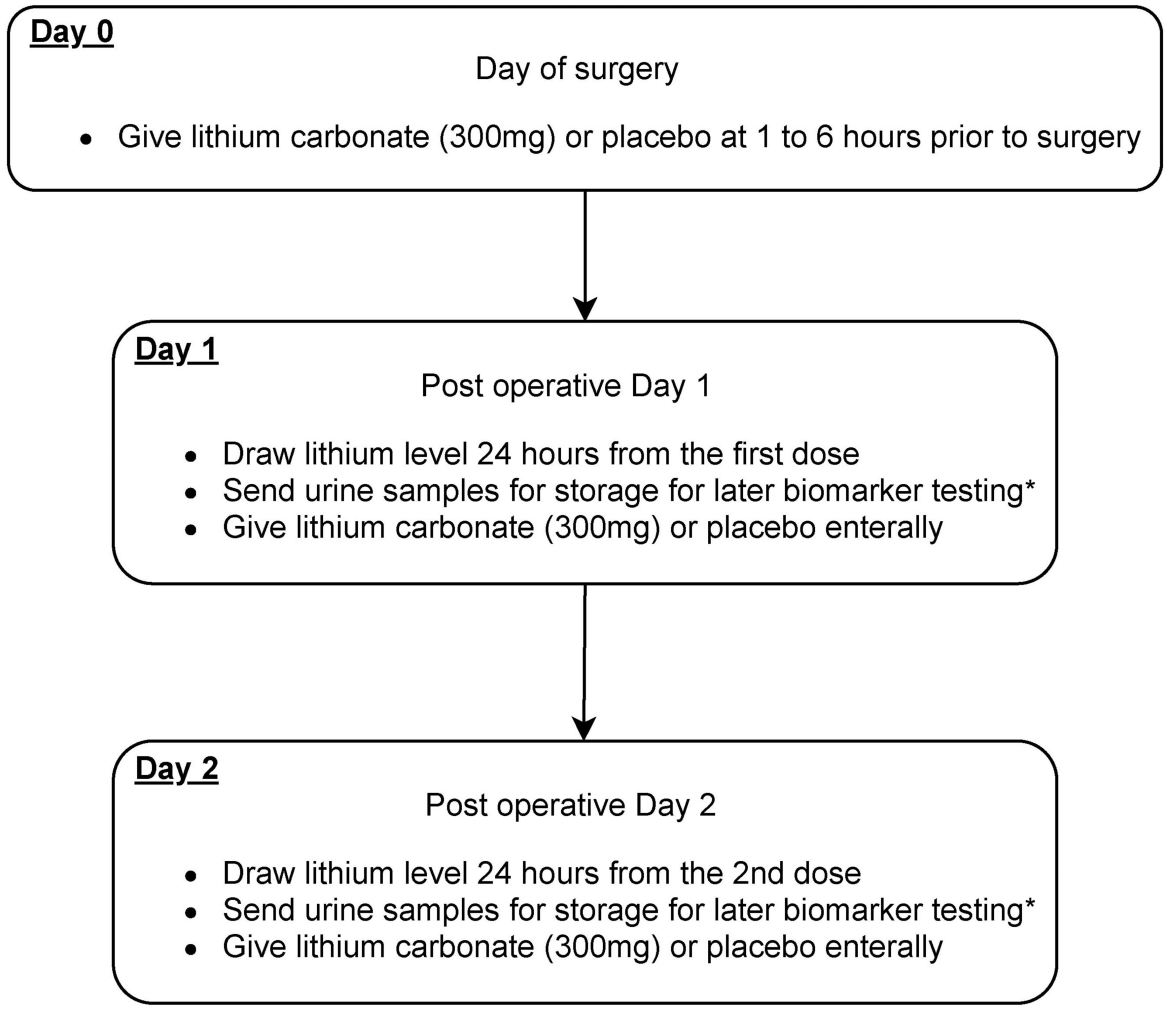
Scheme diagram depicting study interventions to be given in this trial. Patients will receive microdose lithium carbonate or placebo treatment on day 0, 1, and 2 after the cardiovascular surgery. *Biomarkers that will be sent include urinary NGAL, KIM1, tissue inhibitor of metalloproteinases-2 (TIMP-2), and insulin-like growth factor-binding protein 7 (IGFBP-7).

The study intervention is the addition of lithium carbonate for 3 days in addition to usual best practice for perioperative cardiothoracic surgery patients (Figures 2, 3). This is an investigator lead trial without industry input. In brief, the consenting patients will take placebo or small dose lithium carbonate (300 mg) (38) orally on the day of surgery and then two more doses every 24 h. This dose of lithium carbonate is only 1/3 to 1/4 of the standard dose for psychiatric disorders (39). This dose was determined based on a number of pre-clinical studies, which suggest that microdose lithium that is one-third to one-fourth of the neurobiological dose is sufficient to block GSK3β in the diseased kidney and prevent AKI in animal models (36). Blood will be drawn on postoperative day 1 of the surgery and urine samples will be collected on postoperative day 0, 1, 2 and 3. Therapeutic drug monitoring of lithium will be done for 2 days to ensure that the blood concentration of lithium is within therapeutic range. The therapeutic range will be 0.6 to 0.8 meq/L (32, 40). As per standard of care, therapeutic drug monitoring of lithium will be initiated 24 h after the first dose (41).

### Study Population

#### Eligibility Criteria

The inclusion criteria for this trial are as follows: both men and women, 18 years and older, will undergo elective cardiovascular procedure such as aortic valve surgery, mitral valve surgery, coronary artery bypass grafting, or any combination of the above procedures. The subjects must have stable renal function with eGFR > 60 ml/min/1.73 m^2^ and creatinine change <0.3 mg/dl in the preceding 1 month prior to scheduled surgery.

The exclusion criteria for this trial are as follows: subjects who

1. Have ejection fraction of <20% prior to surgery
2. Have estimated glomerular filtration rate <15 ml/min/1.73 m2 as calculated by Chronic Kidney Disease Epidemiology Collaboration (CKD-EPI) formula
3. Will have cardiac surgery performed without using cardiopulmonary bypass
4. Have ongoing sepsis or history of sepsis in the last 2 weeks, defined as having 2 of the following criteria: body temperature >38°C or <36°C, pulse rate >90/min, RR >20/min, WBC >12 or >10% polymorphonuclear cells plus a documented source
5. Have documented rise in creatinine ≥ 0.3 mg/dl in the preceding 1 month prior to surgery
6. Have been deemed to have AKI at the time of the surgery by treating clinician
7. Are on acute or chronic maintenance dialysis at the time of randomization
8. Have AKI due to glomerulonephritis, thrombotic microscopic angiopathy, obstructive nephropathy, interstitial nephritis
9. Are taking lithium for any conditions
10. Unable to absorb lithium or take any medications orally.

### Study Endpoints

The primary endpoint of AKI is a composite of the occurrence of any of the following at 1-week from surgery: AKI based on the Acute Kidney Injury Network (AKIN) criteria. Other components include new onset dialysis at 30 or 90 days from surgery, and death within 90 days in lithium treated patients matched with controls.

Secondary endpoints of renal function/injury, post-operative therapy and infection will be monitored during the patient's hospital stay. Measures of renal function/injury include SCr, albumin to creatinine ratio, estimated glomerular filtration rate (eGFR), and urinary and urinary biomarkers; additionally, the number of days on renal replacement therapy (RRT), days on post-surgical mechanical ventilation, and infection will also be assessed as secondary outcomes.

### Randomization

This will be done by pharmacy according to block technique in 1:1 ratio to either lithium or placebo. Subjects and health care providers will be blinded to the allocation.

### Participant Withdrawal and Follow Up Consent

Subjects will be allowed to drop out of study anytime and if they decide to drop out of the study without completion, they will be asked if collection and analysis of their data could be continued.

### Safety Monitoring and Risk to Subjects

The risk for participants to take low dose lithium in short term is minimal and may include increased urine output, increase in serum sodium, and thirst. This will be explained to patients. Lithium has been used for mental disorders for decades in a dose up to 2,400 mg per day. Hence we do not anticipate that giving lithium for 3 days at a small dose will cause significant toxicity or harm to any patient. However, we will still monitor serum lithium levels to be certain of safety. There is no risk to matched controls who will only receive placebo treatment and standard of care as per cardiothoracic unit protocol. We will have an independent monitor for the study who will be a clinician not involved in recruitment and will be receiving serum lithium levels. The monitor will have access to patients' information who have been recruited for the study. If any provider involved or study monitor feel a safety concern at any time this will be promptly addressed.

### Data Collection

Demographic data (age, sex, race, ethnicity), comorbids, type of surgery, bypass time, aortic cross clamp time, complete blood count, renal function, electrolytes, lithium levels, urinalysis, urine protein and microalbumin, urine testing for AKI biomarkers such as neutrophil gelatinase-associated lipocalin (NGAL), kidney injury molecule-1 (KIM1), tissue inhibitor of metalloproteinases-2 (TIMP-2), and insulin-like growth factor-binding protein 7 (IGFBP7), [TIMP-2] × [IGFBP-7], use of renal replacement therapy type and days, days on mechanical ventilation, infections and death. Source of data is chart review of medical notes, laboratory tests and data will be collected for up to 60 days after surgery. Patients will be followed up in clinic at 6 months. Data will be collected into a web-based database designed to capture all visit information including medical history, results from laboratory analysis and adverse events. Baseline data and ongoing data collection as outlined in Table 1 will be obtained.

**Table 1:** Clinical and laboratory data to be collected at various time points.

Baseline data at screening	Vital signs
	Complete blood count
	Serum chemistry
	^*^eGFR (CKD-EPI creatinine)
	Urinalysis
Perioperative and postoperative data	Vitals signs, intake and output
	Complete blood count
	Serum chemistry, renal function, estimated GFR
	Peak creatinine
	Novel AKI biomarkers: e.g. urinary TIMP-2, IGFBP7, and [TIMP-2] × [IGFBP-7]
	Days on mechanical ventilation
	Days on renal replacement therapy
	Sepsis episodes
	Death
Clinic visit at 6 months	Serum chemistry
	^*^eGFR (CKD-EPI creatinine)

* *Estimated Glomerular filtration rate (eGFR), chronic kidney disease- EPI creatinine (CKD-EPI) equation*.

### Renal Function Measurement

Baseline SCr is defined as the lowest Scr found in the last 3 months prior to encounter with cardiac surgery team. If there is not any prior value available and SCr remains stable at the time of patient encounter, then it will be deemed as baseline SCr. AKI will be defined as an abrupt (within 48 h) reduction in kidney function currently defined as an absolute increase in serum creatinine of more than or equal to 0.3 mg/dl (≥ 26.4 μmol/l), a percentage increase in serum creatinine of more than or equal to 50% (1.5-fold from baseline), or a reduction in urine output (documented oliguria of <0.5 ml/kg per hour for more than 6 h) (42). Patients will be deemed to have CSA-AKI if AKI occurs within 1 week of surgery. Studies have shown that the pattern of AKI recovery influenced the subsequent development of chronic kidney disease (CKD) (43). Thus we will also measure renal function at 6 months post-surgery to see if patients recovered from AKI.

### Data Management

Each patient will be given a unique study identifier. Data will be entered in redcaps by research assistants. The analysis will be done by SAS statistical software. Data will be monitored by data safety monitoring board. The patient identifiers will be kept in a separate, secure index and data collection will be done securely with the index as reference. Patients privacy will be protected as per Health Insurance Portability and Accountability Act protocol.

### Statistical Analysis

This randomized two-arm study is designed to test the null hypothesis of no difference in the primary endpoint of incidence of AKI as defined by AKIN criteria between the two treatment arms, placebo-control and lithium treatment. The primary endpoint will be analyzed on an intention-to-treat basis and patients will therefore be analyzed according to the assigned treatment group at study enrollment. The hypothesis of the study is based on the proportional incidence of AKI as follows:

$$\begin{array}{l} {\text{H}}_{\text{o}}:\lambda_{\text{lithium}}=\;\lambda_{\text{control}}\\ {\text{H}}_{\text{a}}:\lambda_{\text{lithium}}<\;\lambda_{\text{control}} \end{array}$$

Descriptive statistics and exploratory data analysis will be undertaken to summarize baseline characteristics between the control group and the lithium treated group to determine homogeneity and to identify potential differences that might be associated with incidence of AKI. Continuous data will be tested for goodness-of-fit to the normal distribution using Shapiro-Wilk test. If not normally distributed, the log-transformation of the data will be assessed for normality. Continuous data will be expressed as mean ± standard deviation (SD) of the untransformed or log-transformed version, or if neither are normally distributed, as median with interquartile range. Categorical data will be presented as frequency and percent. Comparisons of continuous data will be evaluated using two-sample *t*-tests or Wilcoxan rank sum test. For categorical variables, the chi-square test or, if frequencies are low (≤ 5), Fisher exact test will be used to compare groups. Statistical significance is defined as a *p*-value < 0.05.

To examine the primary endpoint and test the study hypothesis for the effect of lithium on the incidence of AKI, contingency table analysis using chi-square will compare the proportion of AKI within the respective treatment groups. A predictive model that identifies baseline characteristics, including treatment assignment, associated with risk of AKI will be developed. Multivariate testing will use linear regression for continuous outcomes and logistic regression for dichotomous outcomes. Analysis comparing the placebo-control and lithium groups on each baseline characteristic, one at a time, using ANOVA with adjustment for age and sex will be performed. An adjusted logistic regression model based on the dichotomous outcome of occurrence or not of AKI will be further developed to identify characteristics significantly related to AKI. Composite endpoint events, consisting of the first occurrence of AKI, new onset dialysis, and cardiovascular/all-cause mortality, will be compared between treatment groups. Using appropriate uni- and multivariate methods, secondary outcome measures will be incorporated including assessments of renal function/injury such as SCr, NAGL, KIM1, urine TIMP-2, IGFBP7 and [TIMP-2] × [IGFBP-7], urine albumin Cr ratio, as well as indicators of post-operative course including days on mechanical ventilation, days on RRT and infection.

Stepwise multivariate linear regression analysis will be utilized to examine the independent predictors of changes in renal function over time. The longitudinal effect of lithium on kidney function will be estimated by applying generalized linear mixed model with time (continuous variable) as a random coefficient. The percent change in SCr, NAGL, KIM1, urine TIMP-2, IGFBP7 and [TIMP-2] × [IGFBP-7], and urine albumin Cr ratio at 1 week, 30 days and 60 days will be evaluated. Other covariates identified by univariate analysis or by stepwise selection with Akike information criteria will be included in the model. Additionally, risk factors such as age, diabetes, history of chronic kidney disease (CKD) and dialysis, duration of CPB, aortic cross clamp time, type of surgery (emergent vs. elective), valvular vs. non-valvular and duration of surgery will be examined. All multivariate models will be tested for interaction among predictors. Due to the problem of multiple comparisons, the multivariate modeling will be cautiously interpreted and inference will be regarded as limited, but will be examined for possible hypothesis-generating evidence owing to the multifactorial nature of CSA-AKI.

The primary endpoint is based on the first occurrence within 1 week from surgery of AKI and other composite events described above, therefore time-to-event analysis is limited in assessing risk due to the 1-week AKI criteria from exposure at time of surgery. The hazard associated with outcomes outside of the 1-week threshold, such as death and new onset dialysis, will be examined using time-to-event analysis. The predicted probability of the binary occurrence of these endpoints will be calculated using logistic regression with adjustment for age, sex, and baseline renal function measures. Model performance will be summarized using receiver operating characteristic analysis. Time-to-event for the mortality and dialysis endpoints will be examined using Kaplan-Meier analysis with log-rank estimates to compare the lithium and control groups. Hazard ratios will be calculated using Cox proportional-hazard model adjusted as described above. Model diagnostics using Cox-Snell residual plots for goodness-of-fit, and the null hypothesis of proportional hazard assumption will be tested. The extended Cox model will be used to test for interaction among predictors and time.

### Sample Size Estimation

To evaluate the effect of the intervention, that is lithium carbonate on improvement of rates of AKI, we will consider rates of AKI in cardiac surgery patients to be about 20% based on prior data (44, 45). A 25% reduction of this rate is considered clinically significant. Cocks and Torgers (46) proposed a sample size calculation for pilot randomized trials based on confidence interval in which the lower limit of one-sided confidence interval (CI) is greater than the clinically significant effect size in order to exclude the estimate that is considered clinically important. Based on the CI approach, a sample size of 182 with 91 in each group is required to produce a one-sided 80% confidence limit that would exclude a 5% difference. The one-sided confidence interval method is being used since we are only interested in proceeding toward a larger trial if there is evidence of efficacy. Because this is a control vs. intervention study design, there is an inherent improvement in the confidence limit since the controls will constrain some of the random error. This sample size agrees with the recommendation of Teare et al. (47), who suggested 60–100 subjects to estimate the event rate in an intervention group and ensures that we will encounter at least one incident at a 99% confidence level (48). Accordingly, a minimum sample size of 58 with 29 in each group is enough to ensure that at least one event would be detected and is a measure of feasibility that the study can detect the clinically significant outcome.

## Discussion

AKI is a devastating complication that can develop after cardiac surgery, it dictates patient outcomes and impacts morbidity and mortality. It has been shown in a number of studies that AKI independently increases the length of hospitalization, impacts utilization of resources and worsens mortality up to 10 years after surgery (14, 49, 50). Patients who develop CSA-AKI are also at increased risk for developing CKD (51). Prior to using RIFLE and AKIN criteria, the definition of AKI was not standardized and hence incidence of AKI has been variable. Based on AKIN and RIFLE stage 2 criteria the reported rates of AKI after cardiac surgery are 4 to 9% (52–54), although according to stage 1 the incidence rises up to 20% (54, 55).

The pathogenesis of AKI during cardiac surgery is complex and likely multifactorial. Since renal biopsies are rarely done unless injury is felt to be secondary to glomerulonephritis, the histopathological studies are limited. Factors relevant to the development of AKI include patient factors such as advanced age, gender, prior heart failure, baseline CKD, chronic obstructive pulmonary disease or diabetes mellitus (22, 56, 57), procedure-related factors such as duration of surgery, duration of CPB, aortic cross clamp time, exogenous vasopressor use, hemodilution, hypothermia, embolism, and duration of surgery (5, 58–60). Moreover valvular surgery, combined surgery and emergency surgery carry a higher risk for AKI (5). Exogenous factors such as medications like non-steroidal anti-inflammatory agents (NSAIDs), diuretics renin angiotensin system blockers (RAS blockers), and radiocontrast could also impair glomerular autoregulation (61, 62). Moreover, genetic predisposition is important too with apolipoprotein epsilon 4 allele being protective while 2 and 3 increase risk of AKI (5, 63). Furthermore, endogenous toxins such as heme or iron also play an important role. Many patients experience perioperative hypoxia that can lead to endothelial injury by various cytokines (64, 65). During manipulation of aorta and cross clamping there is risk of embolization of gas, debris and atherosclerotic plaque (66, 67). Besides, CPB results in systemic inflammatory immune response due to contact between blood and artificial surface of the CBP circuit. This can lead to release of various cytokines and free radicals (68, 69). The renal medulla is especially sensitive to free radical damage as it lives in a state of perpetual hypoxia (70). All these factors lead to AKI in susceptible patients.

Many efforts have been made to try to mitigate CSA-AKI. For instance, a small prospective study was done to evaluate beneficial effect of thoracic epidural anesthesia compared to general anesthesia leading to slightly reduced incidence of AKI (2 vs. 6.9%) (71). Remote ischemic preconditioning (RIPC) is thought to have a protective effect on distant organs *via* immunomodulation, humoral and neurogenic pathways. It has been shown in meta-analysis that there is a trend for reduction of AKI by using RIPC (72). Besides, an intra-aortic filter has been used to reduce risk of embolization. These atheroma filtration machines have not been able to prevent AKI but have led to improved cognition post-coronary artery bypass grafting surgery (73). In addition, diuretics have been tried for preventing AKI but have shown to worsen the disease (74, 75). In smaller studies, fenoldopam seemed to show some promise. It activates adenylate cyclase and inhibits sodium-potassium ATPasa leading to vasodilation and natriuresis in the proximal tubule and loop of henle. However, a large randomized controlled trial showed no difference in renal replacement therapy and trial was stopped early due to futility (76). In another small study, atrial natriuretic peptide showed reduced risk of renal replacement therapy at 21 days after cardiac surgery. However it causes hypotension (77, 78). Prophylactic hemodialysis has also been tried by Durmaz et al. but it was found to increase morbidity and mortality (79). The mainstay for CSA-AKI is really prevention and supportive therapy. Hence, there remains an opportunity for developing novel therapeutics to prevent or treat CSA-AKI.

Failure of clinical trials of new therapies for CSA-AKI is due, at least in part, to lack of a sensitive and specific marker for AKI. SCr is not a reliable marker for AKI. It is influenced by muscles mass, protein intake, volume status. Urine output also depends on confounding factors such as diuretics use, fluid administration and others. Hence, it is plausible that if we rely only on urine output or SCr we may miss some patients with relatively more short lived AKI, fluctuating volume status or low muscle mass. Some new biomarkers have been characterized in CSA-AKI and have shown to be helpful in the detection of early AKI (80). Urine TIMP-2 and IGFBP7 are new biomarkers for AKI and perform better than existing markers. They are both inducers of G1 cell-cycle arrest. In a study of 42 patients undergoing cardiac surgery, 38% developed AKI. Urinary [TIMP-2]·[IGFBP7] was found to be a sensitive and specific marker of AKI after cardiac surgery (81). In another study of patients undergoing CPB surgery the urinary [TIMP-2]·[IGFBP7] ratio was not able to detect AKI in the early postoperative period but did identify AKI patients on the first post-operative day (82). Based on existing evidence, we will also apply the NephroCheck test, an FDA-approved immunoassay test that measures the urinary concentrations of [TIMP-2] × [IGFBP-7], and measure urinary [TIMP-2]·[IGFBP7] on post-operative day 0, 1 and 2.

Recently, more and more data suggest that GSK3β plays a detrimental role in the pathogenesis of AKI in animal models exposed to nephrotoxic substance or subjected to renal ischemia/reperfusion injury or endotoxemia. Therapeutic targeting of GSK3β by genetic knockout or various selective chemical inhibitors has a demonstrable benefit in a number of pre-clinical studies of AKI. The underlying mechanism has been attributed to mitigated renal tubular cell apoptosis, diminished renal inflammation, boosted antioxidant response, and enhanced renal tubular repair. Among the many selective inhibitors of GSK3β, lithium is of particular interest because it has been safely used in patients with mental diseases as a FDA-approved mood stabilizer for over 50 years (83). However, in order to achieve an actionable concentration in the brain, the ionized lithium needs very high dose to pass through the blood-brain barrier, and produce therapeutic effects in psychiatric disorders. At the high psychiatric dose of lithium for long term, some adverse effects can be elicited in peripheral organs including the kidney. While the exact “kidney-protective dose” of lithium is still unknown, latest evidence from animal data suggests that a small dose of lithium for short term unequivocally exerts a potent renoprotective effect in multiple pre-clinical models of AKI, including nephrotoxic AKI, septic AKI and ischemic AKI (36). Even a delayed treatment with microdose lithium could successfully protect against AKI and promote kidney repair and recovery of kidney function (35). All existing evidence infers that short term microdose lithium therapy may be repurposed for preventing AKI.

In summary, opportunities exist in the field of AKI for newer, efficacious therapies. It is promising that low dose lithium has potential to promote tubular regeneration and hence may ameliorate AKI due to acute tubular necrosis in the setting of cardiac surgery. Successful completion of this pilot study will help to assess the effectiveness of lithium in CSA-AKI. If we find a significant difference in the intervention group, this will pave the way for large full scale randomized trials to further evaluate the potential of this novel regimen.

## Author Contributions

SS, PB, BC, and RG drafted the manuscript. SS is the study coordinator and responsible for patient recruitment, data collection and management. BC prepared the table and figures. PB and TC are the biostatisticians responsible for data management and analyses. SS, LD, and RG participated in study design, data management, and data analysis. All authors reviewed and edited the manuscript and approved the final version.

## Conflict of Interest

The authors declare that the research was conducted in the absence of any commercial or financial relationships that could be construed as a potential conflict of interest.

## Abbreviations

AKI: acute kidney injury
AKIN: acute kidney injury network
CKD: chronic kidney disease
CKD-EPI: chronic kidney disease epidemiology collaboration
CPB: cardiopulmonary bypass
CSA-AKI: cardiac surgery associated acute kidney injury
SCr: serum creatinine
CI: confidence interval
eGFR: estimated glomerular filtration rate
FDA: food and drug administration
GSK3β: glycogen synthase kinase
LiCS-AKI: lithium in cardiac surgery-associated acute kidney injury
RRT: renal replacement therapy
TIMP-2: tissue inhibitor of metalloproteinases-2
IGFBP7: insulin-like growth factor-binding protein 7
ROC: receiver operating characteristic
RIPC: remote ischemic preconditioning.
